# The bZIP transcription factor MdHY5 regulates anthocyanin accumulation and nitrate assimilation in apple

**DOI:** 10.1038/hortres.2017.23

**Published:** 2017-06-07

**Authors:** Jian-Ping An, Feng-Jia Qu, Ji-Fang Yao, Xiao-Na Wang, Chun-Xiang You, Xiao-Fei Wang, Yu-Jin Hao

**Affiliations:** 1 National Key Laboratory of Crop Biology, MOA Key Laboratory of Horticultural Crop Biology and Germplasm Innovation, College of Horticulture Science and Engineering, Shandong Agricultural University, Tai’An 271018, Shandong, China; 2 College of Life Science, Shandong Agricultural University, Tai’An 271018, Shandong, China

## Abstract

The basic leucine zipper (bZIP) transcription factor HY5 plays a multifaceted role in plant growth and development. Here the apple *MdHY5* gene was cloned based on its homology with *Arabidopsis HY5*. Expression analysis demonstrated that *MdHY5* transcription was induced by light and abscisic acid treatments. Electrophoretic mobility shift assays and transient expression assays subsequently showed that MdHY5 positively regulated both its own transcription and that of *MdMYB10* by binding to E-box and G-box motifs, respectively. Furthermore, we obtained transgenic apple calli that overexpressed the *MdHY5* gene, and apple calli coloration assays showed that *MdHY5* promoted anthocyanin accumulation by regulating expression of the *MdMYB10* gene and downstream anthocyanin biosynthesis genes. In addition, the transcript levels of a series of nitrate reductase genes and nitrate uptake genes in both wild-type and transgenic apple calli were detected. In association with increased nitrate reductase activities and nitrate contents, the results indicated that MdHY5 might be an important regulator in nutrient assimilation. Taken together, these results indicate that *MdHY5* plays a vital role in anthocyanin accumulation and nitrate assimilation in apple.

## Introduction

Apple (*Malus*×*domestica*) is an important fruit crop that is widely cultivated worldwide. Peel color is an important trait that determines apple market value; red fruits are more attractive to consumers. As a kind of secondary metabolite, anthocyanins are responsible for the red coloration in apple peel.^1,2^ In general, anthocyanins are synthesized via the phenylpropanoid pathway, and multiple enzymes are involved in this pathway, including *phenylalanine ammonia lyase*, *chalcone isomerase*, *chalcone synthase*, *flavanone 3-hydroxylase* (*F3H*), *dihydroflavonol 4-reductase* (*DFR*), *UDP glucose: flavonoid 3-O-glucosyltransferase* (*UF3GT*) and *anthocyanidin synthase* (*ANS*).^3,4^

Three main protein families—MYB, bHLH and WD40—are involved in the regulation of anthocyanin biosynthesis by forming the MYB-bHLH-WD40 protein complex.^5,6^ In apple, three MYB transcription factors (TFs) (MdMYB1, MdMYB10 and MdMYBA) have been functionally identified and confirmed to be responsible for anthocyanin accumulation, as these TFs directly regulate the expression of anthocyanin biosynthesis structural genes.^7,8^

In addition to genetic components, nutritional components such as nitrogen, phosphorus and potassium can affect fruit coloration as well as plant growth.^9,10^ Among these nutritional components, nitrogen is the most important; NO_3_^−^ and NH_4_^+^ are the major sources of nitrogen in aerobic and anoxic soils, respectively.^11^ In recent years, the physiological process and genetic mechanism of nitrogen uptake and transport have been studied in depth, and protein families, such as NRT1/2, NAR2 and AMT1/2, participate in the absorption and transport of NO_3_^−^ and NH_4_^+^.^12,13,14,15^ In addition, NO_3_^−^ is widely regarded as a crucial signal molecule that modulates multiple aspects of plant growth and development, such as nitrate-mediated root development,^16,17^ stress tolerance^18^ and crop yield and quality.^19,20^ Therefore, identification of the functions of genes involved in nitrogen uptake and transport in apple is essential.

TFs are a group of key regulatory proteins that play important roles in controlling the expression of signal response genes. They can be divided into many families, such as the basic leucine zipper (bZIP), bHLH, MYB, zinc-finger and NAC families, according to their conserved domains. In addition, different types of TFs play diverse roles in plant growth and development. An important TF in plants is the bZIP protein HY5, which was first identified to positively regulate plant photomorphogenesis based on the light insensitivity of the *hy5* mutant.^21,22^ This factor has also been subsequently implicated in abscisic acid (ABA) signaling.^23^

HY5 acts as a master regulator that integrates signals from multiple pathways to coordinate plant stress tolerance and development.^24^ HY5 binds the T/G-box (CACGTT), E-box (CAATTG), GATA-box (GATGATA), ACE-box (ACGT), Z-box (ATACGGT) and C-box (GTCANN) as well as the hybrid C/G- (G) and C/A-boxes in the promoters of many genes that are involved in various signaling pathways, such as light signaling,^25^ anthocyanin and chlorophyll biosynthesis,^26,27^ nutrient signaling^28^ and defense signaling.^29^ In addition, HY5 regulates the expression of microRNAs by directly binding to their promoters,^30^ which indicates that HY5 regulates gene expression at both the transcriptional and post-transcriptional levels. The HY5 protein is unstable and is degraded in darkness by COP1 via the 26S proteasome pathway,^31,32^ but HY5 stability is indirectly promoted in blue light by SPA1 by dissociating with COP1 and associating with CRY1.^33^ Moreover, recent studies have shown that low-temperature and short-heat shock treatments also stabilize the HY5 protein.^26,34^ Thus, HY5 is dynamic in plants and plays a central role in the hub of transcriptional networks.

In the present study, we cloned and functionally characterized the apple bZIP protein MdHY5. MdHY5 may be involved in anthocyanin biosynthesis by directly activating *MdMYB10* and nitrogen signaling by promoting the expression of nitrate reductase (NR) genes and nitrate uptake genes. In brief, these results indicate that MdHY5 plays a vital role in anthocyanin accumulation and nitrate assimilation in apple.

## Materials and methods

### Plant materials and growth conditions

Calli of the ‘Orin’ apple (wild type) were subcultured on Murashige and Skoog (MS) medium containing 0.5 mg L^−1^ indole acetic acid and 1.5 mg L^−1^ 6-butyric acid (BA) at room temperature (24 °C) in continuous darkness; calli were subcultured at 20 days intervals. For high-light and low-temperature treatments, transgenic and wild-type (WT) apple calli were transferred to a phytotron at 15 °C under constant high light (photon flux density of ~100 μmol s^−1^ m^−2^).

For light treatment, apomictic crabapple (*Malus hupehensis*) seedlings grown in darkness for 3 days at room temperature were transferred to white light conditions. For ABA treatment, apomictic crabapple seedlings were treated with 50 μM ABA. RT-qPCR and semi-quantitative RT-PCR were performed to monitor the expression level of *MdHY5*.

### Gene cloning of *MdHY5*

Using the NCBI database with the Basic Local Alignment Search Tool (BLAST) program, we found two potential homologous genes (MDP0000586302 and MDP0000264514) that differed by only one amino acid. On the basis of these results, we speculate that these two genes might have similar functions and possibly be allelic. MDP0000586302 was selected for functional identification.

### RT-qPCR and semi-quantitative RT-PCR

Apple seedlings and 4 g of apple calli collected from three plates were used for RNA extraction. RNAs were extracted from the apple seedlings and calli using RNA plant plus Reagent (Tiangen, Beijing, China) followed by reverse transcription using a PrimeScript first-strand cDNA synthesis kit (Takara, Dalian, China). RT-qPCR assays were conducted with the UltraSYBR mixture (SYBR Green I) (Takara) using an ABI7500 RT-qPCR system. The concentration of cDNA was diluted to 1–10 ng μL^−1^. One microliter of diluted cDNA was used for RT-qPCR, and the 2^−ΔΔCt^ calculation method of RT-qPCR was used. The results were normalized to those of *MdACTIN*. A minimum of three biological replicates per sample were used for RT-qPCR.

RT-PCR was conducted to examine the expression levels of *MdHY5* in response to light and ABA. Each PCR mixture contained 200 ng of cDNA, 1× Taq buffer, 2.5 mM dNTPs, 0.5 μL of Taq DNA polymerase (Trans, Beijing, China) and each primer at 10 μm in a total volume of 25 μL. The reactions were performed as follows: denaturation at 95 °C for 10 min; 25–28 cycles at 95 °C for 30 s, 58 °C for 30 s and 72 °C for 30 s; and a final cycle at 72 °C for 5 min.

The transcription levels of *MdHY5*, anthocyanin biosynthesis genes and nitrogen signaling-related genes (MdNIA1: MDP0000585462; MdNIA2: MDP0000280001; MdNRT1.1: MDP0000163192; MdNRT1.5: MDP0000260604; MdNRT1.7: MDP0000681768; MdNRT2.1: MDP0000239537; MdNRT2.4: MDP0000897809; MdNRT2.5: MDP0000266497; and MdNRT2.7: MDP0000131368) were examined using specific primers.^35^ All of the primers used are shown in Supplementary Table 1.

### Amino-acid sequence analysis and phylogenetic tree construction

The Protein BLAST program (http://www.ncbi.nlm.nih.gov/BLAST/) was used to obtain homologs of *Arabidopsis* HY5. The amino-acid secondary structure of MdHY5 was predicted using the Simple Modular Architecture Research Tool (SMART) software program (http://smart.embl-heidelberg.de/).

The phylogenetic tree was constructed using MEGA 5.0 software.

### Plasmid construction and genetic transformation of apple calli

The overexpression vector *MdHY5-pCAMBIA1300* was constructed by inserting the *MdHY5* open reading frame into the transformed vector pCAMBIA1300.

To generate *MdHY5* transgenic apple calli, the recombinant plasmid was introduced into *Agrobacterium tumefaciens* LBA4404 as described by An *et al.*^36^ Regarding the transformation of apple calli, 15-day-old ‘Orin’ calli (WT) were co-cultured with *Agrobacterium* carrying *MdHY5-*pCAMBIA1300. The calli were co-cultured on MS medium containing 1.5 mg L^−1^ 2, 4-dichlorophenoxyacetic acid and 0.5 mg L^−1^ 6-butyric acid for 2 days at room temperature. The calli were then washed three times with sterile water and transferred to selective media supplemented with 300 mg L^−1^ carbenicillin and 35 mg L^−1^ hygromycin for transgene selection. The transgenic apple calli were co-cultivated in selective media that contained appropriate concentrations of antibiotics.

### Measurement of anthocyanins

Total anthocyanins were extracted as described by Lee and Wicker.^37^ Three grams of apple calli were incubated in an anthocyanin extraction solution for 12 h at 4 °C in darkness. The absorbance of each sample was measured at 530, 620 and 650 nm using a spectrophotometer (Shimadzu UV-2450, Kyoto, Japan). The relative anthocyanin contents were normalized according to the following formula: optical density (OD)=(A530−A620)−[0.1×(A650−A620)]. One unit of anthocyanin content was expressed as a change of 0.1×OD (units×10^3^ g^−1^ of fresh weight (FW)). Measurements were performed in triplicate.

### MdHY5-GST fusion protein expression and purification

The open reading frames of *MdHY5* were cloned into the pGEX-4T-1 vector, which contained a glutathione (GST) tag sequence, and the recombinant vector was then transformed into *Escherichia coli* BL21 (DE3). The BL21 bacteria were subsequently treated with 3 mM isopropyl β-D-1-thiogalactopyranoside to induce the production of the MdHY5-GST fusion protein.

The fusion protein was then added to a cobalt chelate affinity resin containing the immobilized GST-tag and incubated at 4 °C for 2 h under rotation. After three washings, the proteins were collected and detected via western blot with GST antibodies (Abcam, Cambridge, UK).

### Electrophoretic mobility shift assay (EMSA)

EMSA was conducted using a LightShift Chemiluminescent EMSA Kit (Thermo, Waltham, MA, USA). Briefly, biotin-labeled probes were incubated in a 1× binding buffer containing 2.5% glycerol, 10 mM EDTA, 5 mM MgCl_2_ and 50 mM KCl with or without proteins at 24 °C for 25 min. An unlabeled probe was added to the reactions for unlabeled probe competition.

### Transient expression assay in *Nicotiana benthamiana* leaves

Transient expression assays in *N. benthamiana* leaves were performed as previously described.^38,39^ The *MdHY5* and *MdMYB10* promoters were amplified and cloned into pGreenII 0800-LUC vectors, which generated the reporter constructs *MdHY5*_*pro*_*:Luc* and *MdMYB10*_*pro*_*:Luc*, respectively. Site-directed mutagenesis was performed using the Takara MutanBEST kit. The effector (*35S*_*pro*_*:MdHY5*) was constructed by cloning the open reading frame of the *MdHY5* into the pGreenII 62-SK vector. A charge-coupled device imaging apparatus (NightOWL II LB983 in conjunction with Indigo software) was used to collect the LUC images and quantify luminescence intensity. Transformed leaves were sprayed with and soaked in 100 mM luciferin, after which they were placed in darkness for 6 min before luminescence examination.

### Measurement of NR activity

NR activity was measured as previously described.^40^ Five-hundred-milligram samples (apple calli) were transferred to test tubes after being washed with distilled water and weighed; 1 mL of trichloroacetic acid served as the control. Then, 9 mL of 0.1 M phosphate buffer (pH=7.5) mixed with 3% propanol and 0.1 M KNO_3_ was added to the tubes, and the samples were vacuum infiltrated until the samples sunk to the bottom of the tubes. The reactions were performed at 30 °C in the dark for 30 min, and 1 mL of trichloroacetic acid was added to stop the reaction. After standing for 2 min, 4 mL of sulfanilamide mixed with 3 M HCl and 4 mL of 0.2% *N*-1-naphthyl-ethylene-diamine were added to the 2 mL of supernatant, which was transferred into a new tube. Finally, after 30 min, the absorbance was measured at 540 nm. Next, 0–2 g of NaNO_2_ per reaction was used to generate a standard curve. A regression equation was calculated according to the standard curve. NR activity is presented as nanomoles of nitrite produced per hour per gram of FW (nmol nitrite h^−1^ g^−1^ FW). Measurements were performed in triplicate.

### Measurement of nitrate content

Nitrate content was measured according to the salicylic acid method.^41,42^ First, samples of ~1 g (apple calli) were frozen in liquid nitrogen and milled into powder. Next, 10 mL of deionized water was added to the tubes. The samples were boiled at 100 °C for 20 min and centrifuged at 15 000 *g* for 10 min, after which 0.1 mL of the supernatant was transferred to a new tube. Then, 0.4 mL of a 5% salicylic acid–sulfuric acid solution was then added to the tubes. The reactions occurred at room temperature for 20 min, after which 9.5 mL of 8% NaOH was slowly added to the reactions. After the samples cooled to room temperature, the optical density at 410 nm (OD_410_ value) was measured; deionized water served as the control. The nitrate content was calculated according to the following equation: N=C×V/W (N, nitrate content; C, nitrate concentration calculated using OD_410_ and the regression equation; V, total volume of extracted sample; W, weight of sample). Concentrations of 10–120 mg L^−1^ KNO_3_ were used to generate a standard curve. The regression equation was calculated according to the standard curve. Measurements were performed in triplicate.

### Statistical analysis

Statistical analysis was conducted as previously described using appropriate methods and R (3.0.2) software with the R Commander package.^43^ Differences were considered statistically significant when *P*<0.05 and *P*<0.01. The results were analyzed in triplicate.

## Results

### Identification of the *HY5* gene in apple

**Figure 1:**
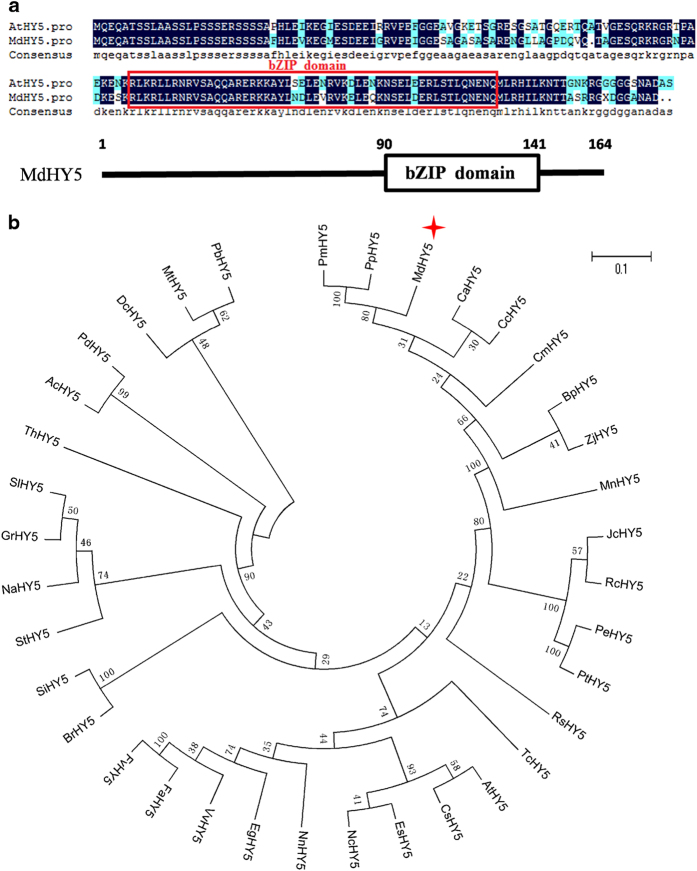
Sequence alignment and phylogenetic analysis of MdHY5. (**a**) Protein alignment of MdHY5 and its homologs in *Arabidopsis*. The zinc-finger domain is boxed. (**b**) Phylogenetic analysis of MdHY5 and 35 other plants HY5 protein sequences obtained from the NCBI database. MdHY5 is denoted by the red asterisk, and the scale bar indicates the branch length. RsHY5: *Raphanus sativus*, XP_018445811.1; AtHY5: *Arabidopsis thaliana*, AT5G11260.1; BrHY5: *Brassica rapa*, XP_009121971.1; ThHY5: *Tarenaya hassleriana*, XP_010541629.1; PeHY5: *Populus euphratica*, XP_011039711.1; MdHY5: *Malus domestica*, MDP0000586302; PbHY5: *Pyrus bretschneideri*, XP_009355719.1; FvHY5: *Fragaria vesca*, XP_004291469.1; ZjHY5: *Ziziphus jujuba*, XP_015885857.1; PmHY5: *Prunus mume*, XP_008219477.1; FaHY5: *Fragaria ananassa*, AKG58815.1; PpHY5: *Prunus persica*, ONI34365.1; CcHY5: *Citrus clementina*, XP_006450470.1; BpHY5: *Betula platyphylla*, AHY20043.1; EgHY5: *Eucalyptus grandis*, XP_010048982.1; JcHY5: *Jatropha curcas*, XP_012076602.1; CaHY5: *Camptotheca acuminate*, APD29065.1; PtHY5: *Populus trichocarpa*, XP_002308656.1; CmHY5: *Cucumis melo*, NP_001284656.1; TcHY5: *Theobroma cacao*, XP_007013841.2; RcHY5: *Ricinus communis*, XP_002515537.1; VvHY5: *Vitis vinifera*, XP_010648648.1; MnHY5: *Morus notabilis*, XP_010110356.1; SiHY5: *Sesamum indicum*, XP_011081579.1; NnHY5: *Nelumbo nucifera*, XP_010250037.1; NcHY5: *Noccaea caerulescens*, JAU18721.1; EsHY5: *Eutrema salsugineum*, XP_006399627.1; StHY5: *Solanum tuberosum*, XP_006361723.1; MtHY5: *Medicago truncatula*, XP_013459310.1; SlHY5: *Solanum lycopersicum*, NP_001234820.1; PdHY5: *Phoenix dactylifera*, XP_008785002.1; AcHY5: *Ananas comosus*, XP_020097860.1; NaHY5: *Nicotiana attenuate*, XP_019265660.1; DcHY5: *Daucus carota*, XP_017229054.1; GrHY5: *Gentiana rigescens*, AIC64080.1; CsHY5: *Camelina sativa*, XP_010419684.1.

Regarding the identification of the *HY5* gene in the apple genome, the *Arabidopsis HY5* gene was used as a query to search similar sequences in apple by mining the NCBI database with the BLAST program. Two potential homologous genes were found: GenBank accession numbers MDP0000586302 and MDP0000264514, which differed by only one amino acid (Supplementary Figure S1). The gene MDP0000586302 was selected for functional identification. This gene contained a 495-bp open reading frame that encoded a protein containing 164 amino acids and was named *MdHY5*. In addition, the protein secondary structure of MdHY5 showed that it contained a bZIP domain on its C-terminal side (amino acids 90–141) (Figure 1a), which indicated the correlation between structure and function as observed in other bZIP proteins.

To analyze the phylogenetic relationship between MdHY5 and HY5 proteins from other plant species, a phylogenetic tree of 36 plant HY5 proteins was constructed using MEGA 5.0. Figure 1b shows that MdHY5 was most closely related to and exhibited the highest homology with PpHY5 and PmHY5 from *Prunus persica* and *Prunus mume*, respectively.

### Expression analysis of the *MdHY5* gene

**Figure 2:**
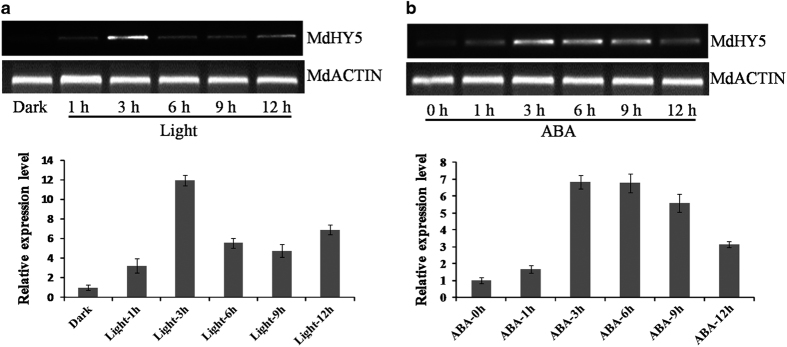
Effects of light and ABA on the transcript level of *MdHY5.* (**a**) Apomictic crabapple (*Malus hupehensis*) seedlings grown in darkness for 3 days at room temperature were treated with continuous white light for 1, 3, 6, 9 and 12 h. The transcript levels of *MdHY5* were determined using RT-PCR (above) and RT-qPCR (below); the value for dark-treated plants was set to 1. (**b**) Apomictic crabapple (*M. hupehensis*) seedlings were treated with 50 μM ABA at the indicated time intervals. The expression levels of MdHY5 were monitored by RT-qPCR (above) and RT-PCR (below); the value for untreated plants was set to 1.

*Arabidopsis HY5* plays a vital role in light signaling and ABA responses.^23,25^ To investigate the functions of MdHY5 *in planta*, the expression patterns of *MdHY5* were determined by RT-qPCR and semi-quantitative RT-PCR using cDNA isolated from apomictic crabapple seedlings treated with light or subjected to ABA. These results demonstrated that the expression levels of *MdHY5* increased in response to light and ABA treatments. The expression level increased 1 h after light treatment and peaked at 3 h (Figure 2a). In addition, treatment of apple seedlings with ABA led to a peak in the expression of *MdHY5* at 3–6 h (Figure 2b). Therefore, MdHY5 plays an important role in light signaling and ABA responses.

### MdHY5 positively regulates its own transcription

**Figure 3:**
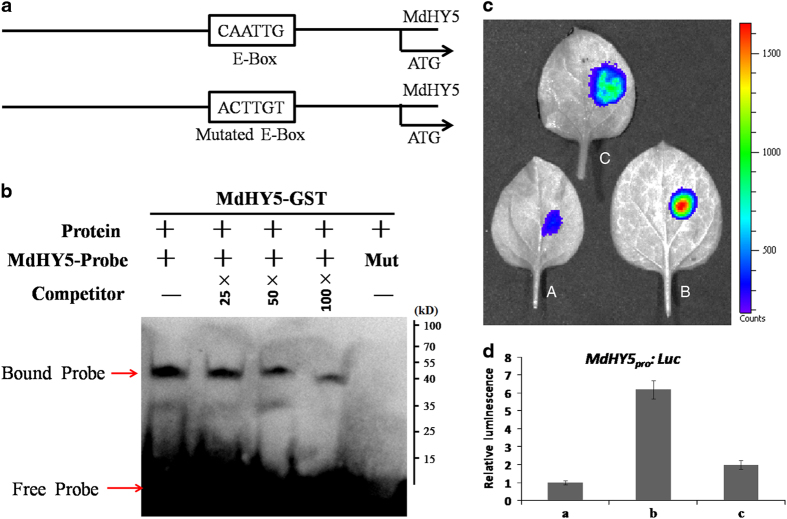
MdHY5 directly and positively regulates its own expression. (**a**) Schematic diagram showing both the predicted E-box and mutated E-box sites in black boxes. (**b**) EMSA results showing that the MdHY5-GST fusion protein directly binds to its own promoter at the E-box *in vitro*. Biotin-labeled probes were incubated with MdHY5-GST protein, and the free and bound DNAs (arrows) were separated in an acrylamide gel. Unlabeled probes served as competitors. The 5′-CAATTG-3′ motif in mutated probes (Mut) was replaced with 5′-ACTTGT-3′. (**c**) Transient expression assays showing that MdHY5 promotes its own expression. A: *MdHY5*_*pro*_*:Luc*; B: *MdHY5*_*pro*_*:Luc*-*35S*_*pro*_*:MdHY5*; C: *MdHY5*_*pro*_*(Mut):Luc*-*35S*_*pro*_*:MdHY5*. Representative images of *Nicotiana benthamiana* leaves 72 h after infiltration are shown. (**d**) Quantitative analysis of luminescence intensity in **c**. The value for *MdHY5*_*pro*_*:Luc* was set to 1.

HY5 and its homolog HYH can regulate *HY5* gene induction in *Arabidopsis*.^44^ To test whether MdHY5 could also directly interact with the *MdHY5* promoter *in vitro*, we expressed and purified recombinant MdHY5-GST fusion protein and prepared *MdHY5* promoter fragments containing versions of the E-box motif. As predicted, EMSAs demonstrated that MdHY5 bound to the oligonucleotide sequence of the CAATTG-box of *MdHY5* (Figures 3a and b).

To investigate whether MdHY5 could activate its own transcription, we carried out a transient assay to compare the activation effect of MdHY5 on the expression of *MdHY5*_*pro*_:*Luc* and *MdHY5*_*pro*_*(Mut):Luc* reporters containing the *MdHY5* promoter fragments or mutated *MdHY5* promoter fragments fused with *LUC* genes. As shown in Figures 3c and d, co-expression of *MdHY5*_*pro*_*:Luc* with *35S*_*pro*_*:MdHY5* markedly increased the luminescence intensity. In contrast, *35S*_*pro*_*:MdHY5* failed to induce the expression of *MdHY5*_*pro*_*(Mut):Luc*. This result suggests that MdHY5 is also capable of positively regulating its own expression.

### MdHY5 promotes anthocyanin accumulation by directly binding to the *MdMYB10* promoter

**Figure 4:**
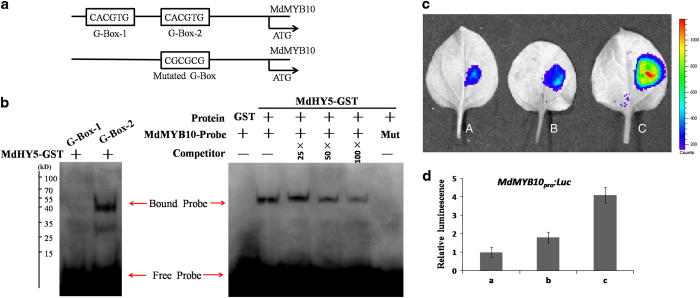
MdHY5 promotes the expression of MdMYB10 by directly interacting with its promoter. (**a**) Schematic diagram of the *MdMYB10* promoter showing the potential MdHY5-binding sites. The predicated two G-box (G-box-1 and G-box-2) and mutated G-box sites and sequences are indicated with black boxes. (**b**) EMSA results showing that MdHY5-GST fusion protein directly binds to the *MdMYB10* promoter at G-box-2 *in vitro*. Biotin-labeled probes were incubated with MdHY5-GST protein, and the free and bound DNAs (arrows) were separated in an acrylamide gel. Unlabeled probes served as competitors. The 5′-CACGTG-3′ motif in mutated probes (Mut) was replaced with 5′-CGTGTG-3′. (**c**) Transient expression assays showing that MdHY5 promotes the expression of *MdMY*B10. A: *MdMYB10*_*pro*_*:Luc*; B: *MdMYB10*_*pro*_*(Mut):Luc-35S*_*pro*_*:MdHY5*; C: *MdMYB10*_*pro*_*:Luc-35S*_*pro*_*:MdHY5*. Representative images of *Nicotiana benthamiana* leaves 72 h after infiltration are shown. (**d**) Quantitative analysis of luminescence intensity in **c**. The value for a *MdMYB10*_*pro*_*:Luc* was set to 1.

*Arabidopsis HY5* promotes anthocyanin biosynthesis by inducing the expression of *PAP1*.^27^ To investigate this in apple, we investigated the DNA–protein interaction between MdHY5 and *MdMYB10* promoter fragments containing G-boxes. As shown in Figure 4a, the upstream region of the *MdMYB10* gene contained two MdHY5-binding sites (G-box-1 and G-box-2). Interestingly, the EMSA assays showed that MdHY5 interacted with an *MdMYB10* promoter fragment (G-box-2) (Figure 4b). In addition, the transient assay suggested that MdHY5 could induce the transcriptional activation of *MdMYB10* in apple.

**Figure 5:**
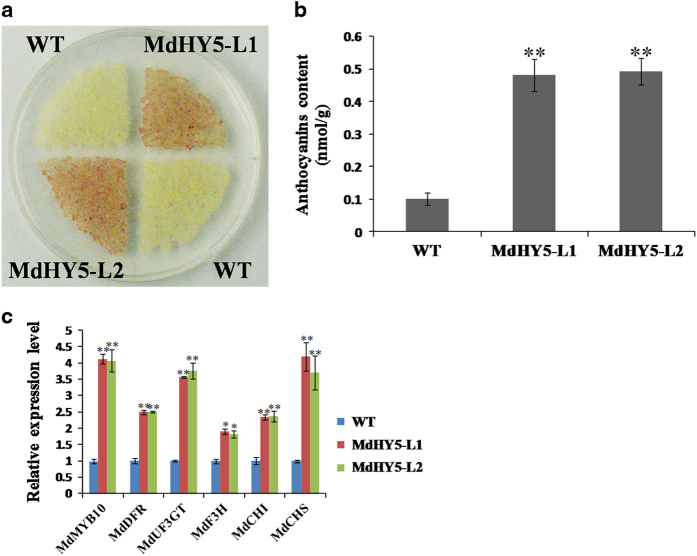
MdHY5 increases anthocyanin accumulation in transgenic apple calli. (**a**, **b**) Anthocyanin contents in transgenic calli (MdHY5-L1 and MdHY5-L2) and wild-type (WT) control grown on medium under low-temperature and high-light conditions. (**c**) Relative expression levels of *MdMYB10*, *MdDFR*, *MdUF3GT*, *MdF3H*, *MdCHI* and *MdCHS* in transgenic calli and WT control. The value for WT was set to 1.

To examine whether *MdHY5* regulates anthocyanin accumulation, we transformed the overexpression construct *MdHY5*-pCAMBIA1300 into ‘Orin’ apple calli through *Agrobacterium*-mediated genetic transformation (Supplementary Figure S2). The observation of the appearance of the apple calli revealed that the *MdHY5* overexpression calli (MdHY5-L1 and MdHY5-L2) appeared redder in color under low-temperature and high-light conditions than did the WT control (Figure 5a). Spectrophotometric analysis demonstrated that the apple calli overexpressing *MdHY5* produced much higher contents of anthocyanins (Figure 5b). In addition, the expression levels of flavonoid structural genes in the WT and transgenic calli were analyzed by RT-qPCR. The overexpression of *MdHY5* significantly upregulated the expression of primary genes involved in the anthocyanin biosynthesis pathway, including *MdMYB10*, *MdDFR*, *MdUF3GT*, *MdF3H*, *MdCHS* and *MdCHI* (Figure 5c). This result suggests that *MdHY5* affects the accumulation of anthocyanins. Taken together, these results demonstrate that MdHY5 binds to the G-box-2 site of the *MdMYB10* promoter to induce anthocyanin biosynthesis in apple.

### MdHY5 is involved in nitrate assimilation

**Figure 6:**
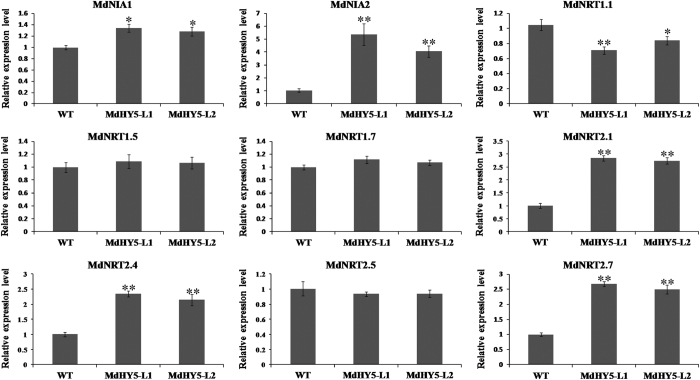
MdHY5 influences the expression of nitrogen signaling-related genes. RT-qPCR analysis of genes related to nitrogen signaling in WT and *MdHY5* transgenic calli (MdHY5-L1 and MdHY5-L2) under low-temperature and high-light conditions. The value for WT was set to 1.

*Arabidopsis* HY5 and HYH upregulate NR genes but negatively regulate nitrate uptake genes.^45^ To examine whether MdHY5 influenced nitrogen signaling in apple, we attempted to identify the possible target genes of MdHY5; we therefore investigated the *cis* elements in the NR genes and nitrate uptake genes in apple. Supplementary Figure S3 shows that four genes related to nitrogen signaling (*MdNIA1*, *MdNRT1.1*, *MdNRT2.4* and *MdNRT2.7*) contained potential MdHY5-binding sites. The expression levels of a series of NR genes and nitrate uptake genes were subsequently detected. As shown in Figure 6, MdHY5 positively regulated the expression of the NR gene *MdNIA2*, which is in agreement with the observation in *Arabidopsis*. In contrast to the results for *MdNIA2*, MdHY5 negatively regulated the expression of the nitrate uptake gene *MdNRT1.1* in apple. In addition, overexpression of *MdHY5* promoted the expression of *MdNRT2.1*, *MdNRT2.4* and *MdNRT2.7*, which might help coordinate the acquisition of plant carbon and nitrogen.^28^

**Figure 7:**
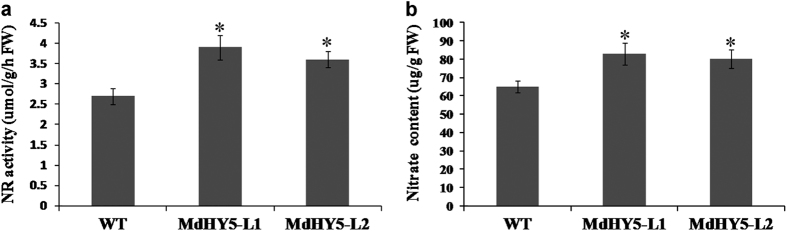
MdHY5 influences NR activity and nitrate acquisition. (**a**) NR activities and (**b**) nitrate contents of WT and *MdHY5* transgenic calli (MdHY5-L1 and MdHY5-L2) under low-temperature and high-light conditions are shown.

We also examined NR activity and nitrate content. As shown in Figure 7a, NR activities increased in *MdHY5*-overexpressing apple calli that were grown under low-temperature and high-light conditions, which indicates that MdHY5 might be a positive regulator of NR in apple. In addition, the nitrate contents of transgenic apple calli markedly increased (Figure 7b), which might be associated with increased demand for metabolites during plant growth.

## Discussion

The bZIP TF HY5 is a central regulator of plant growth and development and has been functionally characterized to be involved in multifaceted developmental processes such as cell elongation and proliferation,^46,47^ chloroplast development,^48,49^ photomorphogenesis, pigment accumulation,^27,50,51, 52^ nutrient assimilation and carbon/nitrogen balance.^28,53^ Other *HY5*-mediated response pathways, such as hormone signaling,^23,54^ reactive oxygen species and defense signaling,^29,55^ as well as temperature responses^26^ have recently been uncovered. Among these responses, pigment accumulation and nutrient assimilation are economically important characteristics that influence apple quality and yield.

Here we cloned and isolated a gene from the apple genome that contains a highly conserved bZIP motif in its C terminus and is highly homologous to HY5 proteins from other species; this finding indicated that MdHY5 might have similar functions as other HY5 proteins. As we predicted, our molecular and genetic analyses implied that MdHY5 was highly similar in structure and function to *Arabidopsis* HY5 in terms of the conserved bZIP domain (Figure 1) and response to light and ABA signaling (Figure 2). In addition, EMSA assays showed that MdHY5 could bind to the E-box motif of its promoter (Figure 3), which suggests a potential autoregulatory loop for *MdHY5* transcription. These results indicate that the functions of MdHY5 are conserved across different species.

To further investigate the functions of MdHY5 *in planta*, we used *Agrobacterium*-mediated transformation to obtain transgenic apple calli overexpressing *MdHY5* under the control of the 35S promoter (Supplementary Figure S2). HY5 is a positive regulator of flavonoid biosynthesis by modulating the expression of *MYB75*/*PAP1* in *Arabidopsis*.^27,30^ We also investigated the role of MdHY5 in the regulation of flavonoid biosynthesis on the basis of EMSA-binding assays and apple calli coloration detection. As hypothesized, the EMSA results showed that MdHY5 was able to bind the G-box site of the *MdMYB10* promoter (Figure 4). Furthermore, the *MdHY5*-overexpressing apple calli produced much higher amounts of anthocyanins than did the WT control, and the RT-qPCR analysis indicated that anthocyanin biosynthesis genes, including *MdDFR*, *MdUF3GT*, *MdF3H*, *MdCHI* and *MdCHS*, were clearly upregulated in the *MdHY5* transgenic lines (Figure 5). These results suggest that MdHY5 promotes anthocyanin accumulation by directly binding to the *MdMYB10* promoter.

In addition, HY5 positively regulates NR activity and coordinates plant carbon and nitrogen acquisition by affecting the expression of both NR genes and nitrate uptake genes in *Arabidopsis*.^28,45,53^ To determine whether MdHY5 is also required for nitrogen assimilation in apple, we measured the transcripts of a series of NR genes and nitrate uptake genes in transgenic and WT apple calli. MdHY5 positively regulated the expression of *MdNIA2*, *MdNRT2.1*, *MdNRT2.4* and *MdNRT2.7* but negatively regulated the expression of *MdNRT1.1*, which was in agreement with observations in *Arabidopsis* (Figure 6).^28,53^ In addition, the upstream region of the *MdNIA1* gene may contain a putative MdHY5-binding site (G-box) (Supplementary Figure S3), and overexpression of *MdHY5* slightly induced the transcription of *MdNIA1*, despite AtHY5 having no impact on the expression of *MdNIA1*.^28^ The function of MdHY5 might be distinct from that of *Arabidopsis* HY5, or the function of MdHY5 might be a secondary metabolic effect caused by the upregulation of *MdNIA2*. In addition, the increased NR activities and nitrate contents in *MdHY5*-overexpressing apple calli might be associated with the increased demand for metabolites during plant growth (Figure 7). Therefore, MdHY5 may play a vital role in nitrogen assimilation in apple. However, these results are not sufficient and require further investigation.

**Figure 8:**
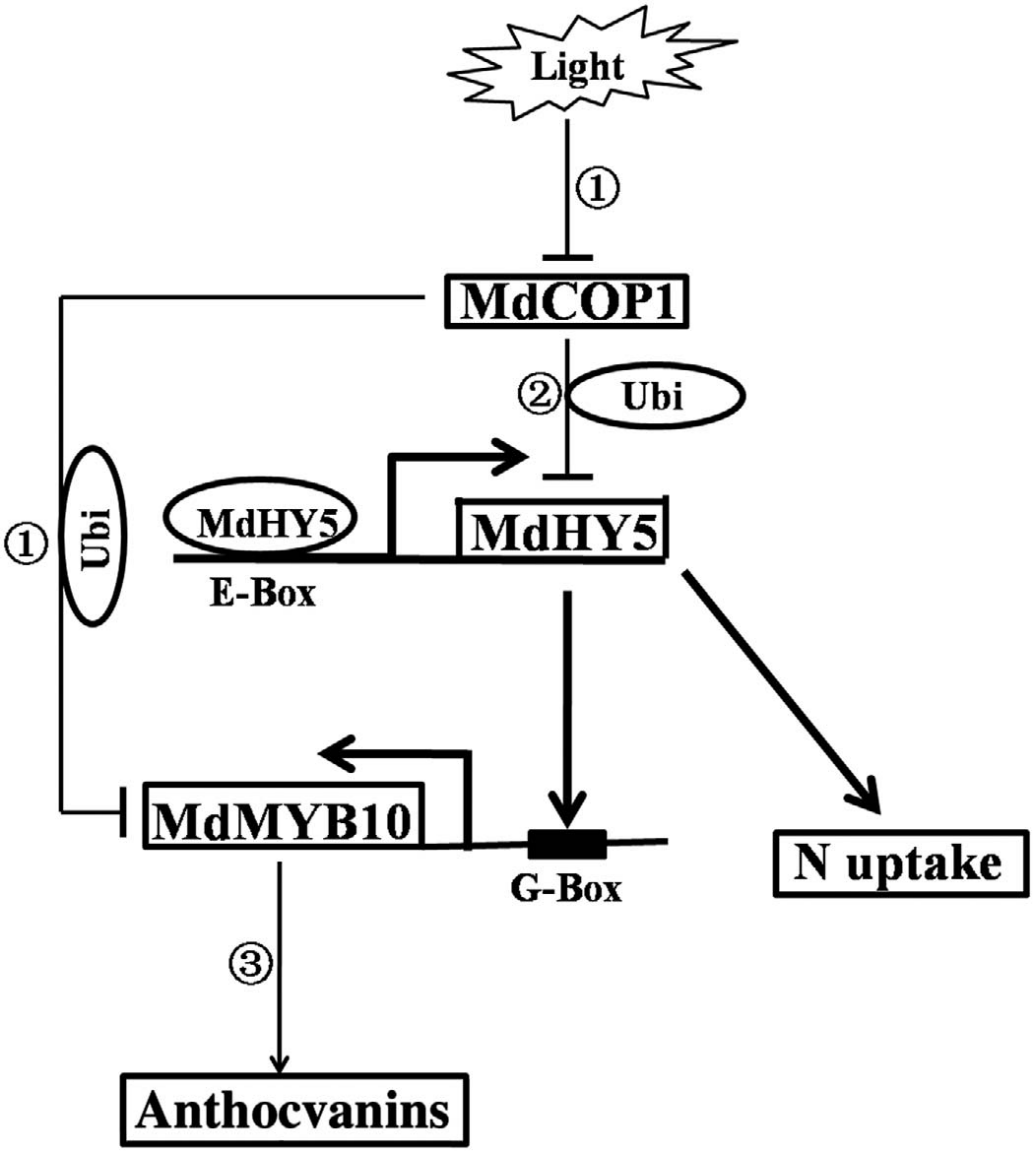
Model of *MdHY5*-mediated anthocyanin accumulation and nitrate assimilation. The bold arrows indicate the pathway verified in apple in the present work. The arrows indicate pathways that have been previously reported in apple or *Arabidopsis*. MdCOP1: apple ubiquitin E3 ligase CONSTITUTIVE PHOTOMORPHOGENIC 1; Ubi: ubiquitination. (1) Li *et al.*^56^ and Maier *et al.*;^57^ (2) Ang *et al.*;^22^ (3) Takos *et al.*^58^ and Ban *et al.*^59^

On the basis of our results and previous studies in model plant species, we propose a hypothetical working model for *MdHY5*-regulated anthocyanin accumulation and nitrate assimilation (Figure 8). MdHY5 is released from MdCOP1-mediated degradation after detection of light signaling. MdHY5 then directly binds to the G-box site of the *MdMYB10* promoter to induce its expression to regulate anthocyanin biosynthesis. In addition, MdHY5 may also be involved in nitrate assimilation by regulating the transcription of both NR genes and nitrate uptake genes. At the same time, MdHY5 induces its own expression in all light conditions by directly binding to the E-box site of its promoter. Improved comprehension of MdHY5 function and signaling in apple could be highly useful for improving fruit traits, such as pigment accumulation and nitrogen use efficiency, for producing high-quality fruits.
