# Supplementary material for: The bZIP transcription factor MdHY5 regulates anthocyanin accumulation and nitrate assimilation in apple
Source: Hortic Res. 2017 Jun 7;4:17023–. doi: 10.1038/hortres.2017.23 (PMC5461414; doi:10.1038/hortres.2017.23)
Supplement: Supplementary Information [file hortres201723-s1.docx]

**
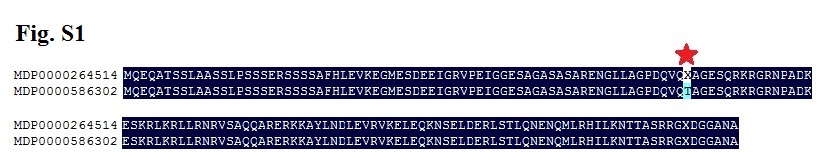
**

**Fig. S1 The sequence comparison of MDP0000586302 and MDP0000264514**

The red mark denoted the amino acid difference.


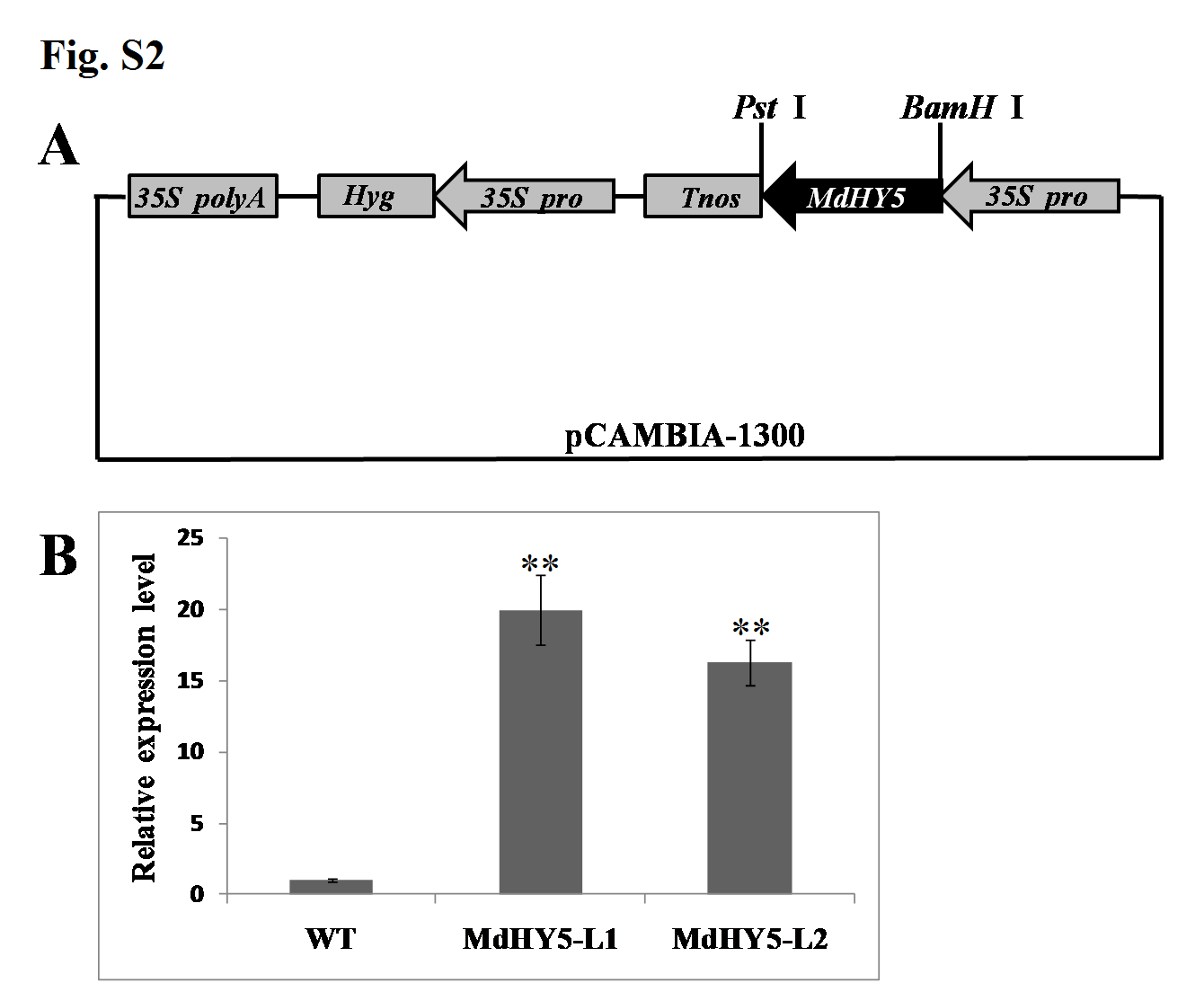


**Fig. S2 Vector construction and generation of transgenic apple calli**

**(A)** Schematic diagram of the 35S promoter: MdHY5 construct. **(B)** RT-qPCR analysis of the expression level of *MdHY5* in MdHY5-L1 and MdHY5-L2 transgenic apple calli

**
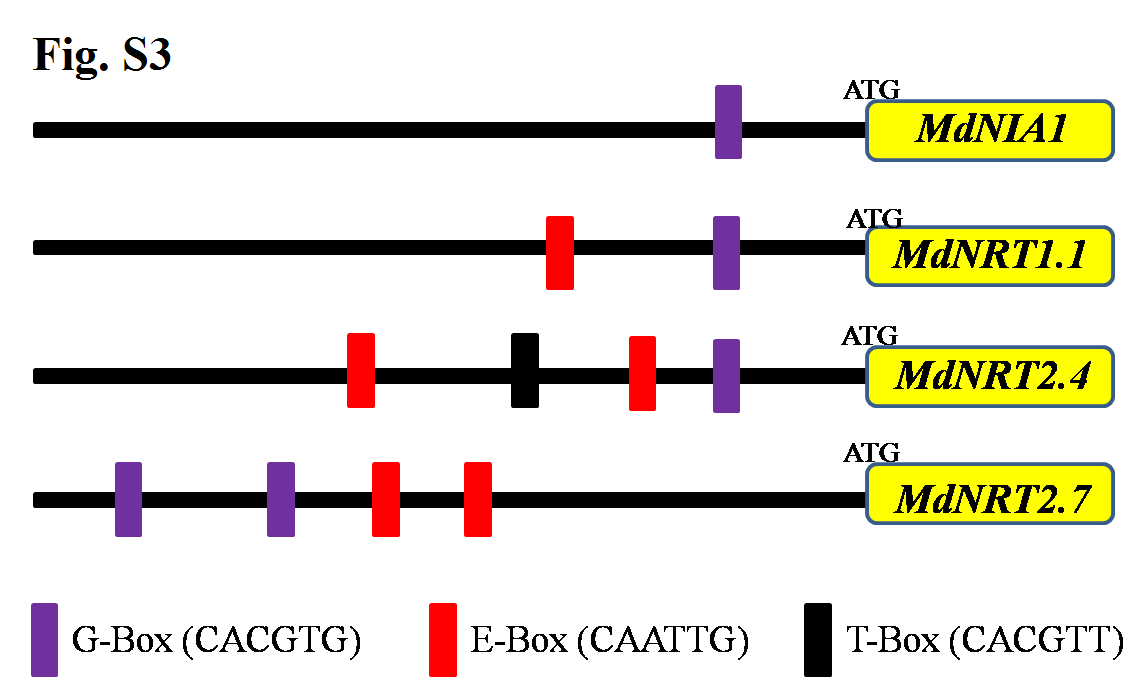
**

**Fig. S3 Analysis of the promoters of nitrogen signaling-related genes**

The T/G-Box and E-Box are marked in the illustration of *cis* elements upstream of the nitrogen signaling-related genes (*MdNIA1*, *MdNRT1.1*, *MdNRT2.4*, *MdNRT2.7*).

**Supplementary Table 1**

Primers used for gene expression analysis and vector construction in this study.

Primer name sequence (5’ to 3’)

MdACTIN-F ACACGGGGAGGTAGTGACAA

MdACTIN-R CCTCCAATGGATCCTCGTTA

MdHY5-F ATGCAAGAGCAGGCGACGAG

MdHY5-R ATCCGCATTTGCACCACCAT

MdHY5(E-box) GTCGAC GAGCCAATTGCGTCGACAG

MdHY5(E-box-mut) GTCGAC GAGCACTTGTCGTCGACAG

MdMYB10(G-box) GTCGAC ACCACGTGGAGTTAAATT

MdMYB10(G-box-mut) GTCGACACCGCGCGGAGTTAAATT

MdHY5(qRT)-F AGAGCAGGCGACGAGCTCCCT

MdHY5(qRT)-R TCTGCTGGATTTCTTCCTCTC

MdMYB1(qRT)-F GAAAGAGCTGCATATCCCAG

MdMYB1(qRT)-R CTATTCTTCTTTTGAATGATTCC

MdDFR(qRT)-F GTTGAGGGAGATAGGGTTTGAG

MdDFR(qRT)-R GGTAAATGTAAAACAATAGAGAGG

MdUF3GT(qRT)-F GGAAGTGGTTTTGTCGCCTG

MdUF3GT(qRT)-R CATTATTATTGAGCAACGAACAGC

MdF3H(qRT)-F GCCGATCACCTACACCGAG

MdF3H(qRT)-R: GTACAAGAAGTGGGAAGGC

MdCHI(qRT)-F GCTACAAATGCGGTGATAG

MdCHI(qRT)-R CGCCTCCACTACAACCTCC

MdCHS(qRT)-F GGCAAGTGCTGTCGGATT

MdCHS(qRT)-R CCCAAAGAAATAACCACAAG

MdNRT1.1(qRT)-F TCCTTTATGCTCTGCTTGCTC

MdNRT1.1(qRT)-R GGGACTGTGGTTGAGATGGT

MdNRT1.5(qRT)-F CCAAGCAAGACATCGGAAAT

MdNRT1.5(qRT)-R GTAGCGTGGCCCTAGCAAAT

MdNRT1.7(qRT)-F GCGTCATCTCCTTTCTCACC

MdNRT1.7(qRT)-R TCCCGATTCCACATCTTTG

MdNRT2.1(qRT)-F AGTCGCTTGCACGTTACCTG

MdNRT2.1(qRT)-R ACCCTCTGACTTGGCGTTCTC

MdNRT2.4(qRT)-F TCCTTCCAGCGTCCAAAGATGT

MdNRT2.4(qRT)-R TGGGAAGGTGTGGTGTTTGGT

MdNRT2.5(qRT)-F TCGATCTGAAACTCCACACG

MdNRT2.5(qRT)-R ACTTCTTCGCCACAACATCC

MdNRT2.7(qRT)-F TAACTCCTTCATCGCCATCC

MdNRT2.7(qRT)-R GACTACGCATCCCGAGAACA

MdNIA1(qRT)-F AACCCGCCGATAAACAGAC

MdNIA1(qRT)-R GTTCGCAGTTGAAGGGATGT

MdNIA2(qRT)-F CGGGAAGAAAGTCACACGAG

MdNIA2(qRT)-R CCAGAAACACCAGCACCAGT
